# Systematic Review of Therapeutic Strategies for Reducing Parkinson’s Disease Symptoms and Progression

**DOI:** 10.7759/cureus.99206

**Published:** 2025-12-14

**Authors:** Srihas Tumu, Venkata Yashashwini Maram Reddy, Navod Jayasuriya, Muaz Ali, Abdaal Munir, Iana Malasevskaia, Jamal Montaser

**Affiliations:** 1 Psychiatry and Behavioral Sciences, California Institute of Behavioral Neurosciences and Psychology, Fairfield, USA; 2 Internal Medicine, Guntur Medical College, Guntur, IND; 3 Anesthesiology and Perioperative Medicine, Faculty of Medicine, University of Ruhuna, Galle, LKA; 4 Medicine, Rawalpindi Medical University, Rawalpindi, PAK; 5 Surgery, College of Physicians and Surgeons Pakistan, Islammabad, PAK; 6 General Surgery, Wirral University Teaching Hospital, Birkenhead, GBR; 7 Obstetrics and Gynecology, Private Clinic 'Yana Alexandr', Sanaa, YEM

**Keywords:** cognitive decline prevention, neurodegenerative disorders, parkinson’s disease (pd), systematic literature review, therapeutic strategies

## Abstract

Parkinson's disease (PD) is a progressive neurodegenerative disorder characterized by motor and non-motor symptoms, including cognitive decline, which plays a significant role in its pathogenesis. This systematic review aimed to evaluate the efficacy of therapeutic strategies in reducing symptoms and potentially slowing the progression of PD. This systematic review followed Preferred Reporting Items for Systematic Reviews and Meta-Analyses (PRISMA) 2020 guidelines. A systematic search was conducted across PubMed, MEDLINE, Europe PMC, EBSCO Open Dissertations, ScienceDirect, and the Cochrane Library for randomized controlled trials (RCTs) published between January 2015 and January 2025. Inclusion criteria focused on RCTs assessing therapeutic interventions aimed at slowing Parkinsonian symptoms and disease progression compared to placebo or standard care. Study quality was appraised using the Cochrane Risk of Bias-2 (RoB-2) tool.

The review identified 15 RCTs that met the inclusion criteria. Interventions, such as exenatide and apomorphine, demonstrated improvements in motor symptoms, as measured by the Movement Disorder Society-Unified Parkinson's Disease Rating Scale (MDS-UPDRS), often coupled with reductions in inflammatory markers like high-sensitivity C-reactive protein (hs-CRP). However, cognitive interventions yielded mixed results, showing benefits only in specific cognitive domains. The safety profiles varied significantly; for example, apomorphine (93% adverse events) exhibited substantially higher rates of adverse events compared to the placebo group (57%). Therapeutic strategies aimed at targeting PD symptoms show promise in managing both motor and non-motor manifestations of the disease. While benefits for cognitive decline are less consistent, these interventions warrant further investigation for their potential to slow overall disease progression. Future research should prioritize larger, multicenter RCTs with longer follow-up, explore combination therapies, and focus on identifying biomarkers for personalized treatment.

## Introduction and background

Parkinson's disease (PD) is a progressive neurodegenerative disorder characterized by the loss of dopaminergic neurons (nerve cells that produce dopamine) in the substantia nigra pars compacta (a specific region in the midbrain), leading to dopamine deficiency, a neurotransmitter crucial for motor control [1].

PD affects more than 1% of the global population over the age of 60 years [2]. This prevalence is increasing due to an aging population, underscoring the growing clinical relevance of the disease. Approximately 40% of patients experience cognitive impairment, which can progress to dementia [3]. The hallmark motor symptoms of PD include bradykinesia (slow movement), rigidity, tremors, and postural instability, while non-motor symptoms, such as cognitive decline, mood disturbances, and autonomic dysfunction, significantly impact patients' quality of life [4].

Although the exact etiology of PD remains unclear, accumulating evidence suggests that neuroinflammation plays a pivotal role in its pathogenesis and progression [2]. Microglial activation, astrocytic changes, and infiltration of peripheral immune cells into the brain have all been observed in Parkinson's disease, suggesting a chronic inflammatory state within the central nervous system. This neuroinflammation is hypothesized to exacerbate neuronal damage and accelerate disease progression [2].

Current treatments for PD, such as levodopa and dopamine agonists, primarily focus on symptomatic relief by replenishing dopamine levels. However, these therapies do not address the underlying neurodegenerative processes and often lose efficacy over time, necessitating the exploration of novel therapeutic strategies [5]. Targeting cognitive decline, a significant non-motor symptom closely linked to neuroinflammation, has emerged as a promising avenue for disease-modifying interventions. These strategies include the use of anti-inflammatory drugs, cytokine inhibitors, gene therapy, and lifestyle modifications such as diet and exercise [6].

This systematic review addresses the critical gap in evidence by evaluating the efficacy and safety of therapeutic interventions that specifically target the mechanisms underlying cognitive decline and neuroinflammation in PD. The primary objective is to evaluate interventions aimed at slowing disease progression by assessing their impact on motor symptoms, non-motor symptoms, and neuroinflammatory markers, compared to standard care or placebo. By synthesizing the available evidence from randomized controlled trials (RCTs), this review seeks to inform clinical practice and guide future research in the development of disease-modifying therapies for Parkinson’s disease.

## Review

Methods

Study Design

This systematic review was conducted in accordance with the Preferred Reporting Items for Systematic Reviews and Meta-Analyses (PRISMA) 2020 guidelines [7]. The protocol for this systematic review was not registered on the International Prospective Register of Systematic Reviews (PROSPERO). This study aimed to investigate the efficacy and safety of therapeutic strategies in slowing the progression of Parkinson’s disease (PD). The guiding research question of this review was framed using the Population, Intervention, Comparison, Outcome (PICO) framework - Population: patients with idiopathic Parkinson’s disease (PD); Intervention: therapeutic strategies targeting mechanisms related to cognitive decline or neuroinflammation; Comparison: placebo, standard care, or other therapeutic interventions; Outcome: reduction in motor/non-motor symptoms, improvement in cognitive function, or slowing of disease progression, measured by validated scales.

Eligibility Criteria

The eligibility criteria were established prior to conducting the review. Inclusion was strictly limited to randomized controlled trials (RCTs) in human participants. The target population was patients diagnosed with idiopathic Parkinson’s disease (IPD), regardless of disease stage; studies focusing solely on drug-induced Parkinsonism were excluded. Interventions included were therapies specifically designed to target mechanisms related to cognitive decline or neuroinflammation (e.g., anti-inflammatory agents, cytokine inhibitors, specific pharmacological interventions). Comparison groups were mandatory (placebo, standard care/usual care, or another active treatment). Outcomes required reporting on at least one of the following domains: motor function (e.g., Movement Disorder Society-Unified Parkinson's Disease Rating Scale {MDS-UPDRS}), non-motor symptoms (e.g., Non-Motor Symptoms Scale {NMSS}), cognitive function (e.g., Montreal Cognitive Assessment {MoCA}, Scales for Outcomes in Parkinson's disease-Cognition {SCOPA-COG}), or neurobiological markers (e.g., high-sensitivity C-reactive protein {hs-CRP}, Dopamine Transporter Scan {DaTscan}). The language of publication was restricted to English, and studies must have been published within the 10-year period from January 1, 2015, to January 31, 2025. Exclusion criteria included all study types other than RCTs (such as reviews or cohort studies), animal studies, preprints, uncompleted trials, and studies with a high risk of bias (RoB) score determined prior to full-text review.

Data Collection

The data collection involved a thorough search through multiple databases, including PubMed, MEDLINE, Europe PMC, EBSCO Open Dissertations, ScienceDirect, and Cochrane Library. This search was completed on January 31, 2025. This search strategy used specific keywords related to each concept within the research question. For neuroinflammation, keywords such as neuroinflammation, “inflammatory processes,” “brain inflammation,” and “neuroimmune response” were used. For cognitive decline, keywords such as “cognitive impairment,” “cognitive dysfunction,” “memory decline,” “neurocognitive disorders,” and dementia were used. For Parkinson’s disease, the keywords that were used were “Parkinson's disease” PD, “Parkinsonian syndromes,” “motor symptoms,” and “non-motor symptoms.” For therapeutic strategies, “treatment strategies,” “pharmacological interventions,” “anti-inflammatory therapies, and “disease-modifying therapies” were used as keywords. Finally, for standard care and placebo, keywords like “standard care,” “placebo-controlled,” “control group,” “comparator group,” and “usual care” were used. In addition, in databases that support Medical Subject Headings (MeSH) terms, corresponding keywords were used. The full details are shown in Table 1.

**Table 1:** The search strategy and database-specific filters applied for systematic literature retrieval across multiple databases.

Database/register	Search strategy	Filters	Number of results before/after inclusion/exclusion criteria filters	Date
PubMed/MEDLINE	(("Parkinson's disease"[Title/Abstract] OR "PD"[Title/Abstract] OR "Parkinsonian syndromes"[Title/Abstract] OR "Motor symptoms"[Title/Abstract] OR "Non-motor symptoms"[Title/Abstract] OR "Parkinson Disease"[MeSH Terms] OR ("parkinson disease/drug therapy"[MeSH Terms] OR "parkinson disease/prevention and control"[MeSH Terms] OR "parkinson disease/rehabilitation"[MeSH Terms] OR "parkinson disease/therapy"[MeSH Terms])) AND ("2015/01/26 00:00":"3000/01/01 05:00"[Date - Publication] AND ("adaptive clinical trial"[Publication Type] OR "case reports"[Publication Type] OR "clinical trial"[Publication Type] OR "clinical trial, veterinary"[Publication Type] OR "controlled clinical trial"[Publication Type] OR "multicenter study"[Publication Type] OR "observational study"[Publication Type] OR "observational study, veterinary"[Publication Type] OR "randomized controlled trial"[Publication Type] OR "randomized controlled trial, veterinary"[Publication Type]) AND ("humans"[MeSH Terms] OR "animals"[MeSH Terms:noexp]) AND "english"[Language] AND "aged"[MeSH Terms]) AND (((((("Neuroinflammation"[Title/Abstract] OR "Inflammatory processes"[Title/Abstract] OR "Brain inflammation"[Title/Abstract] OR "Cytokines"[Title/Abstract] OR "Neuroimmune response"[Title/Abstract] OR ("Neuroinflammatory diseases"[MeSH Terms] OR "Cytokines"[MeSH Terms])) AND ("2015/01/26 00:00":"3000/01/01 05:00"[Date - Publication] AND ("adaptive clinical trial"[Publication Type] OR "case reports"[Publication Type] OR "clinical trial"[Publication Type] OR "clinical trial, veterinary"[Publication Type] OR "controlled clinical trial"[Publication Type] OR "multicenter study"[Publication Type] OR "observational study"[Publication Type] OR "observational study, veterinary"[Publication Type] OR "randomized controlled trial"[Publication Type] OR "randomized controlled trial, veterinary"[Publication Type]) AND ("humans"[MeSH Terms] OR "animals"[MeSH Terms:noexp]) AND "english"[Language] AND "aged"[MeSH Terms])) OR (("Cognitive impairment"[Title/Abstract] OR "cognitive dysfunction"[Title/Abstract] OR "Memory decline"[Title/Abstract] OR "Neurocognitive disorders"[Title/Abstract] OR "Dementia"[Title/Abstract] OR "Dementia"[MeSH Terms] OR "cognitive dysfunction"[MeSH Terms] OR ("cognitive dysfunction/drug therapy"[MeSH Terms] OR "cognitive dysfunction/prevention and control"[MeSH Terms] OR "cognitive dysfunction/rehabilitation"[MeSH Terms] OR "cognitive dysfunction/therapy"[MeSH Terms])) AND ("2015/01/26 00:00":"3000/01/01 05:00"[Date - Publication] AND ("adaptive clinical trial"[Publication Type] OR "case reports"[Publication Type] OR "clinical trial"[Publication Type] OR "clinical trial, veterinary"[Publication Type] OR "controlled clinical trial"[Publication Type] OR "multicenter study"[Publication Type] OR "observational study"[Publication Type] OR "observational study, veterinary"[Publication Type] OR "randomized controlled trial"[Publication Type] OR "randomized controlled trial, veterinary"[Publication Type]) AND ("humans"[MeSH Terms] OR "animals"[MeSH Terms:noexp]) AND "english"[Language] AND "aged"[MeSH Terms]))) AND ("2015/01/26 00:00":"3000/01/01 05:00"[Date - Publication] AND ("adaptive clinical trial"[Publication Type] OR "case reports"[Publication Type] OR "clinical trial"[Publication Type] OR "clinical trial, veterinary"[Publication Type] OR "controlled clinical trial"[Publication Type] OR "multicenter study"[Publication Type] OR "observational study"[Publication Type] OR "observational study, veterinary"[Publication Type] OR "randomized controlled trial"[Publication Type] OR "randomized controlled trial, veterinary"[Publication Type]) AND ("humans"[MeSH Terms] OR "animals"[MeSH Terms:noexp]) AND "english"[Language] AND "aged"[MeSH Terms])) OR (((("standard care"[Title/Abstract] OR "Placebo-controlled"[Title/Abstract] OR "control group"[Title/Abstract] OR "comparator group"[Title/Abstract] OR "usual care"[Title/Abstract] OR ("reference standards"[MeSH Terms] OR "standards"[MeSH Subheading] OR "control groups"[MeSH Terms] OR "population groups"[MeSH Terms] OR "social group"[MeSH Terms])) AND ("2015/01/26 00:00":"3000/01/01 05:00"[Date - Publication] AND ("adaptive clinical trial"[Publication Type] OR "case reports"[Publication Type] OR "clinical trial"[Publication Type] OR "clinical trial, veterinary"[Publication Type] OR "controlled clinical trial"[Publication Type] OR "multicenter study"[Publication Type] OR "observational study"[Publication Type] OR "observational study, veterinary"[Publication Type] OR "randomized controlled trial"[Publication Type] OR "randomized controlled trial, veterinary"[Publication Type]) AND ("humans"[MeSH Terms] OR "animals"[MeSH Terms:noexp]) AND "english"[Language] AND "aged"[MeSH Terms])) OR 12[UID]) AND ("2015/01/26 00:00":"3000/01/01 05:00"[Date - Publication] AND ("adaptive clinical trial"[Publication Type] OR "case reports"[Publication Type] OR "clinical trial"[Publication Type] OR "clinical trial, veterinary"[Publication Type] OR "controlled clinical trial"[Publication Type] OR "multicenter study"[Publication Type] OR "observational study"[Publication Type] OR "observational study, veterinary"[Publication Type] OR "randomized controlled trial"[Publication Type] OR "randomized controlled trial, veterinary"[Publication Type]) AND ("humans"[MeSH Terms] OR "animals"[MeSH Terms:noexp]) AND "english"[Language] AND "aged"[MeSH Terms]))) AND ("2015/01/26 00:00":"3000/01/01 05:00"[Date - Publication] AND ("adaptive clinical trial"[Publication Type] OR "case reports"[Publication Type] OR "clinical trial"[Publication Type] OR "clinical trial, veterinary"[Publication Type] OR "controlled clinical trial"[Publication Type] OR "multicenter study"[Publication Type] OR "observational study"[Publication Type] OR "observational study, veterinary"[Publication Type] OR "randomized controlled trial"[Publication Type] OR "randomized controlled trial, veterinary"[Publication Type]) AND ("humans"[MeSH Terms] OR "animals"[MeSH Terms:noexp]) AND "english"[Language] AND "aged"[MeSH Terms])))	English 10 years, 40+ years, case reports, controlled clinical trial randomized controlled trial, veterinary adaptive clinical trial, clinical study, equivalence trial, clinical trial, controlled clinical trial, evaluation study, multicenter study, observational study, pragmatic clinical trial	322/52 results	January 24, 2025
Europe PMC	(Neuroinflammation OR “Inflammatory processes” OR “Brain inflammation” OR Cytokines OR “Neuroimmune response”) AND (“Cognitive impairment” OR “Cognitive dysfunction” OR “Memory decline” OR “Neurocognitive disorders” OR Dementia) AND (“Parkinson's disease” OR PD OR “Parkinsonian syndromes” OR “Motor symptoms” OR “Non-motor symptoms”) AND (“Treatment strategies” OR “Pharmacological interventions” OR “Anti-inflammatory therapies” OR “Disease-modifying therapies”) AND (“Standard care” OR Placebo-controlled OR “Control group” OR “Comparator group” OR “Usual care”)	Research articles 10 years	2122/476 results	January 27, 2025
EBSCO Open Dissertations	(Neuroinflammation OR “Inflammatory processes” OR “Brain inflammation” OR Cytokines OR “Neuroimmune response”) AND (“Cognitive impairment” OR “Cognitive dysfunction” OR “Memory decline” OR “Neurocognitive disorders” OR Dementia) AND (“Parkinson's disease” OR PD OR “Parkinsonian syndromes” OR “Motor symptoms” OR “Non-motor symptoms”) AND (“Treatment strategies” OR “Pharmacological interventions” OR “Anti-inflammatory therapies” OR “Disease-modifying therapies”) AND (“Standard care” OR Placebo-controlled OR “Control group” OR “Comparator group” OR “Usual care”)	English 10 years	21/17 results	January 31, 2025
ScienceDirect	(Neuroinflammation OR “Brain inflammation”) AND (Cognitive dysfunction OR Dementia) AND (“Parkinson's disease” OR PD) AND (“Treatment strategies” OR “Pharmacological interventions”) AND (“Standard care”)	Research article 10 years	31/1	January 31, 2025
Cochrane Library	(Neuroinflammation OR “Inflammatory processes” OR “Brain inflammation” OR Cytokines OR “Neuroimmune response”) AND (“Cognitive impairment” OR “Cognitive dysfunction” OR “Memory decline” OR “Neurocognitive disorders” OR Dementia) AND (“Parkinson's disease” OR PD OR “Parkinsonian syndromes” OR “Motor symptoms” OR “Non-motor symptoms”) AND (“Treatment strategies” OR “Pharmacological interventions” OR “Anti-inflammatory therapies” OR “Disease-modifying therapies”) AND (“Standard care” OR Placebo-controlled OR “Control group” OR “Comparator group” OR “Usual care”)	English 10 years	11/11	January 31, 2025

**Figure 1:**
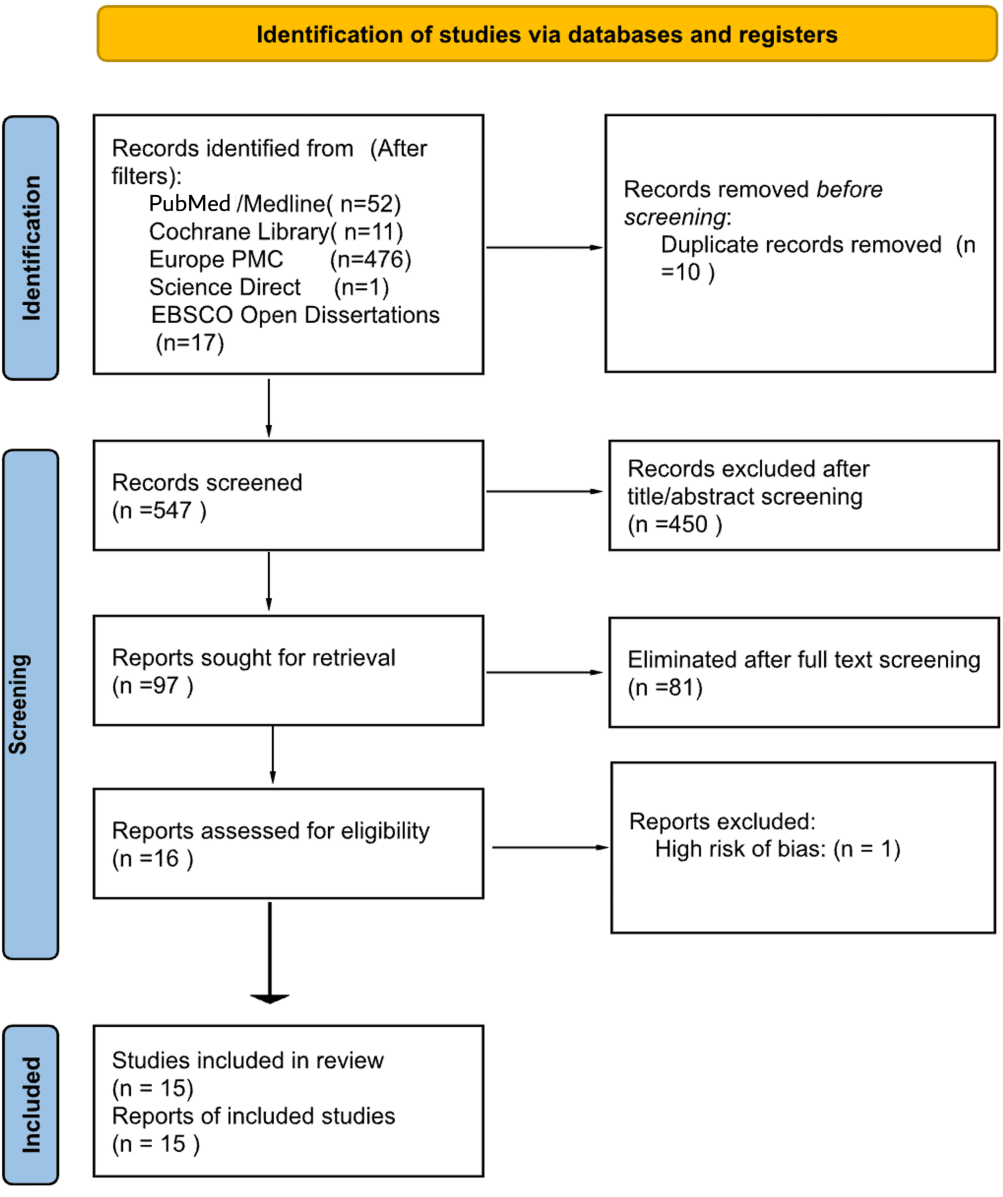
PRISMA flow diagram. PRISMA: Preferred Reporting Items for Systematic Reviews and Meta-Analyses

The search strategy used these various concepts to allow a precise retrieval of related articles. After the initial search, all results were exported to the reference management software Rayyan (Cambridge, MA: Rayyan Systems, Inc.), where duplicates were identified and removed [8]. The remaining titles and abstracts were then screened for eligibility against the pre-defined inclusion and exclusion criteria. This initial screening was followed by a full-text review of all studies that met the initial eligibility criteria. Any disagreements during the screening process were resolved through consensus and discussion with a senior research investigator. The detailed flow of the study selection is documented in the PRISMA flow diagram (Figure 1).

Data Extraction and Quality Assessment

The data extraction portion was performed using a pre-piloted, standardized data extraction form to collect comprehensive information from the included studies. Data extracted included as follows: study design, participant population characteristics (e.g., age, disease duration, Hoehn and Yahr stage), intervention and comparison details, follow-up duration, key findings related to primary and secondary outcomes, and adverse events. To ensure the reliability of this data, the methodological quality of the included studies was evaluated using the Cochrane risk of bias-2 (RoB-2) tool for all included randomized controlled trials (RCTs) [9]. Studies rated as having an overall high risk of bias were excluded prior to the final synthesis. Data were extracted in duplicate by two independent reviewers using a standardized, pre-piloted data extraction form. Discrepancies were reconciled by consensus or by appeal to the third reviewer.

Data Synthesis

Due to the significant clinical and methodological heterogeneity observed across the included interventions (which ranged from pharmacological agents to non-pharmacological approaches) and the variety of outcome measures (e.g., MDS-UPDRS, MoCA, hs-CRP), a quantitative meta-analysis was not performed. Instead, a narrative synthesis was conducted, summarizing the findings from the studies to provide a descriptive overview of the various therapeutic strategies that influenced the progression of Parkinson’s disease (PD). The key results from each study were organized into tables, which allowed for a clear and comparative understanding of the existing evidence.

Results

Study Selection

The systematic search identified a total of 2,470 records. After applying database-specific filters for publication date (2015-2025), English language, and study type (RCTs), a total of 557 records were transferred to the reference management software Rayyan for screening, as detailed in Table 1 [8].

Following the transfer, 10 duplicates were identified and removed, leaving 547 unique records for title and abstract screening. During this initial screening phase, 450 articles were excluded primarily due to a non-randomized controlled trial (RCT) design, an irrelevant population, or an intervention outside the defined scope.

Consequently, 97 articles were carried forward and sought for full-text retrieval. After a thorough full-text assessment, 81 studies were eliminated. One study was excluded due to a rating of high risk of bias following quality appraisal. Ultimately, 15 studies met all criteria and were included in this systematic review. The complete study selection process is illustrated in the PRISMA flow diagram (Figure 1).

Quality Appraisal

The methodological quality of the included studies was assessed using the Cochrane RoB-2 tool [9]. The appraisal revealed low risk of bias in randomization across all studies. However, three studies showed some concerns in domains related to deviations from intended interventions and adherence. Overall, most studies had low risk concerning missing outcome data and consistently assessed outcomes. Notably, one study was found to have a high risk of bias in the measurement of outcomes and was excluded from future review. The selection of reported results was largely free from bias, with clear reporting of pre-specified outcomes (Table 2).

**Table 2:** Risk of bias assessment of included RCT studies using the Cochrane risk of bias-2 (RoB-2) tool. The Cochrane RoB-2 tool assesses five domains. Domain 1: bias arising from the randomization process; domain 2: bias due to deviations from intended interventions; domain 3: bias due to missing outcome data; domain 4: bias in measurement of the outcome; domain 5: bias in selection of the reported result(s). Each domain is scored either (a) low risk (🟢), (b) some concerns (🟡), or (c) high risk (🔴). RCT: randomized controlled trial

Studies	Domain 1	Domain 2	Domain 3	Domain 4	Domain 5	Overall
Yonguc et al. (2019) [2]	🟢	🟢	🟢	🟢	🟢	🟢
Tamtaji et al. (2019) [3]	🟢	🟢	🟢	🟢	🟢	🟢
Athauda et al. (2017) [10]	🟢	🟢	🟢	🟢	🟢	🟢
Katzenschlager et al. (2018) [11]	🟢	🟢	🟢	🟢	🟢	🟢
Tröster et al. (2016) [12]	🟢	🟡	🟢	🟡	🟢	🟡
Peball et al. (2020) [13]	🟢	🟢	🟢	🟢	🟢	🟢
Weintraub et al. (2016) [14]	🟢	🟢	🟢	🟢	🟢	🟢
Mamikonyan et al. (2015) [15]	🟢	🟢	🟢	🟢	🟢	🟢
Mestre et al. (2020) [16]	🟢	🟢	🟢	🟢	🟢	🟢
Murata et al. (2015) [17]	🟢	🟢	🟡	🟢	🟢	🟡
Simuni et al. (2015) [18]	🟢	🟢	🟢	🟢	🟢	🟢
Rieu et al. (2018) [19]	🟢	🟢	🟢	🟢	🟢	🟢
Trung et al. (2019) [20]	🟡	🟢	🟢	🟢	🟢	🟡
Villafane et al. (2017) [21]	🟢	🟢	🟢	🟢	🟢	🟢
Hannink et al. (2019) [22]	🟢	🟡	🟢	🔴	🟢	🔴
Hauser et al. (2016) [23]	🟢	🟢	🟢	🟢	🟢	🟢

Summary of Included Studies

This review includes 15 RCTs evaluating various pharmacological interventions for Parkinson’s disease. The reviewed studies assess various pharmacological and non-pharmacological interventions for Parkinson’s disease, targeting motor and non-motor symptoms. Exenatide, probiotics, apomorphine, safinamide, opicapone, and levodopa-based therapies showed improvements in motor symptoms and "off" time reduction. Cognitive interventions, such as rivastigmine and rasagiline, had mixed results, with some benefits in executive function and motor scores but no strong cognitive effects. Non-motor symptoms, including excessive drooling, overactive bladder, sleep disturbances, and neuropsychiatric issues, were addressed with glycopyrrolate, fesoterodine, rotigotine, and nabilone, showing varying degrees of efficacy. Subthalamic deep-brain stimulation affected cognition, with worsened fluency but improved mood (Table 3). Overall, these interventions provide targeted symptom relief, but their effects vary depending on disease stage and patient characteristics.

**Table 3:** Summary of the included studies. CI: confidence interval; CFU: colony forming units; UI: units; VAS: visual analog scale; SD: standard deviation; P: probability, used in statistics to show how likely a result will be; hs-CRP: high-sensitivity C-reactive protein; GSH: glutathione; MDA: malondialdehyde; HOMA-IR: Homeostatic Model Assessment for Insulin Resistance; QUICKI: Quantitative Insulin Sensitivity Check Index; CSF: cerebrospinal fluid; PD: Parkinson's disease; nOH: neurogenic orthostatic hypotension; MDS-UPDRS: Movement Disorder Society-Unified Parkinson's Disease Rating Scale; LEDD: levodopa equivalent daily dose; DaTscan: dopamine transporter scan; PGIC: patient global impression of change; SCOPA-COG: Scales for Outcomes in Parkinson's Disease-Cognition; MoCA: Montreal Cognitive Assessment; PDAQ-15: Parkinson's Disease Activities Questionnaire-15; UPDRS: Unified Parkinson's Disease Rating Scale; ADCS-CGIC: Alzheimer's Disease Cooperative Study-Clinical Global Impression of Change; DRS-2: Dementia Rating Scale-2; PDQ-8: Parkinson's Disease Questionnaire-8; PDQ-39: Parkinson's Disease Questionnaire-39; ROMP-Saliva: Royal Prince Alfred Hospital Medical Centre Sialorrhea Scale; SSS: Sialorrhea Scoring Scale; SEADL: Schwab and England Activities of Daily Living Scale; GDS-15: 15-item Geriatric Depression Scale; PFTD: painful foot tonic dystonia; BFM: Burke-Fahn-Marsden Scale; DLPFC: dorsolateral prefrontal cortex; MMSE: Mini-Mental State Examination; SEAPI: severity of emotional and physical symptoms of incontinence; ICIQ-SF: International Consultation on Incontinence Questionnaire-Short Form; OAB-V8: Overactive Bladder-V8 Symptom Score; Mattis DRS: Mattis Dementia Rating Scale; CGI-I: Clinical Global Impression of Improvement; NMSS: Non-Motor Symptoms Scale; DRS: Dementia Rating Scale; WMS-III-A: Wechsler Memory Scale Third Edition-Associates; iTBS: intermittent theta burst stimulation; TMS: transcranial magnetic stimulation

Studies	Population characteristics/sample size	Intervention	Comparison	Duration of treatment/follow-up	Key findings
Yonguc et al. (2019) [2]	Parkinson's disease with overactive bladder symptom, Hoehn and Yahr Stage 1.0-4.0, Ages 40+ years, 63 patients	Fesoterodine fumarate 4 mg daily	Placebo	Four weeks of double-blind treatment. One week washout. Four weeks of open-label treatment	Non-motor: fesoterodine significantly reduced micturition episodes vs. placebo (p<0.001). Significant improvements in nocturia, urgency, and quality of life (QoL) measures. Cognition (MMSE): stable in both groups
Tamtaji et al. (2019) [3]	People with Parkinson's disease. 60 patients enrolled (30 in the probiotic group, 30 in the placebo group), ages 50-90 years	Probiotic administration (8×10^9^ CFU/day) containing: Lactobacillus acidophilus, Bifidobacterium bifidum, Lactobacillus reuteri, Lactobacillus fermentum for 12 weeks	Placebo	12 weeks	Motor: significant improvement in MDS-UPDRS, total score in probiotic group vs. placebo (p=0.01). Biomarkers: significant reduction in inflammatory markers (hs-CRP, MDA) and improvement in insulin sensitivity (p<0.002)
Athauda et al. (2017) [10]	Idiopathic Parkinson’s disease, at Hoehn and Yahr stage 2.5 or less. Sixty-two patients enrolled (32 in exenatide group, 30 in placebo group), ages 25-75 years	Subcutaneous exenatide 2 mg once weekly for 48 weeks	Placebo group receiving matched placebo injections	60 weeks total (48 weeks of treatment + 12-week washout period)	Motor: exenatide group showed significant sustained improvement in MDS-UPDRS part 3 (Off-medication) vs. placebo at 60 weeks (p=0.03). Imaging: DaTscan suggested protection against dopaminergic neuron loss. Cognition: no significant group difference (MDRS)
Katzenschlager et al. (2018) [11]	Parkinson’s disease with motor fluctuations, 107 patients, ages 30+ years	Subcutaneous apomorphine infusion: about 16 h a day	Placebo infusion: saline solution	12 weeks	Motor: significant decrease in "off" time (mean change = -2.47 h, p=0.0025) and increase in "on" time without troublesome dyskinesia (p=0.0008). Improved Patient Global Impression of Change (PGIC) score.
Tröster et al. (2016) [12]	Parkinson’s disease, 136 patients, ages 41-78 years.	101 patients bilateral subthalamic deep-brain stimulation - right after implantation of device.	35 patients had the device implanted, but the stimulation was turned on three months later	12 months	Cognition: significant worsening in executive function (fluency, Stroop scores) at 12 months (p<0.05). Non-motor: significant improvement in mood (Hamilton Depression Inventory, p<0.05)
Peball et al. (2020) [13]	Parkinson’s disease with non-motor symptoms, 47 patients (38 randomized), ages 30+ years	Nabilone 0.25 mg once a day, gradually increasing in steps of 0.25 mg to 2 mg a day	Placebo pill	Four weeks in the second, randomized phase	Non-motor: fewer patients felt worse in the Nabilone group (CGI-I, p=0.049). Nabilone trended toward less worsening in NMSS and MDS-UPDRS-I vs. Placebo
Weintraub et al. (2016) [14]	Parkinson’s disease with mild cognitive impairment, 170 patients, ages 45-80 years	Rasagiline 1 mg per day	Placebo pill	24 weeks	Motor: significant improvement in UPDRS part III (motor) and part II (ADLs) vs. placebo (p<0.02). Cognition (SCOPA-COG): small but significant improvement in SCOPA-COG score vs. Placebo. MoCA stable.
Mamikonyan et al. (2015) [15]	Parkinson’s disease with mild cognitive impairment, 28 patients, ages 45-80 years	Rivastigmine transdermal patch. Patients started with 4.6 mg/24 h for the first four weeks, increased to 9.5 mg/24 h for the remaining six weeks, unless the lower dose was not tolerated	Placebo patch	24 weeks (10 weeks of treatment with each patch, separated by a four-week washout period)	Cognition: significant improvement on the everyday cognition battery (p=0.03). Trend favoring rivastigmine on clinical global change (ADCS-CGIC, p=0.096)
Mestre et al. (2020) [16]	Parkinson’s disease with excessive drooling, 48 patients, mean age: 71.1 years.	Glycopyrrolate	Placebo liquid medicine	12 weeks	Non-motor: glycopyrrolate significantly reduced sialorrhea-related disability (ROMP-Saliva) and severity (SSS) vs. placebo (p<0.05).
Murata et al. (2015) [17]	Parkinson’s disease with wearing-off symptoms, 389 patients, ages 20-74 years	Zonisamide: two different doses were used: 25 mg/day and 50 mg/day.	Placebo pill	12 weeks of active treatment, following a four-week placebo run-in period	Motor: zonisamide 50 mg/day significantly reduced "off" time vs. placebo (p<0.05). The 25 mg dose was not statistically significant
Simuni et al. (2015) [18]	Early idiopathic Parkinson's disease, Hoehn and Yahr score 2 or less. 210 patients, ages 30+ years	72 patients: pioglitazone 15 mg/day, 67 patients: pioglitazone 45 mg/day	Placebo	44 weeks	Motor/primary outcome: primary outcome (change in total UPDRS score) was not met; no significant difference found between either dose of pioglitazone and placebo
Rieu et al. (2018) [19]	Idiopathic Parkinson's disease. Unilateral or bilateral (PFTD) for >1 h/day, 45 patients, ages 30-80 years	Incobotulinum toxin A (Xeomin) 100 UI injected into either the flexor digitorum longus or flexor digitorum brevis. Two injection sessions, 12 weeks apart	Placebo	Assessments at six and 18 weeks after the initial injection	Non-motor: significant improvement in Clinical Global Impression (CGI) of change vs. placebo (p=0.039). Dystonia severity and pain improved significantly vs. baseline
Trung et al. (2019) [20]	Idiopathic Parkinson's disease. Hoehn and Yahr scale stages I to III. Twenty-eight patients, ages 62-80 years	Intermittent theta burst stimulation (iTBS) over the left dorsolateral prefrontal cortex (DLPFC)	Sham stimulation	Neuropsychological assessments (tests of thinking skills) were done before the TMS, and then on days one, 10, and 30 after the last TMS session	Cognition: active iTBS showed significant improvement in overall cognition composite Z-scores at 30 days vs. baseline (p=0.011). Significant gains in attention and visuospatial abilities
Villafane et al. (2017) [21]	Parkinson's disease for at least three years. Responded well to L-DOPA medication. Had some motor fluctuations in 40 patients, median age 70 years	Transdermal nicotine patches. Dose gradually increased to 90 mg/day (or highest tolerated dose) over 11 weeks. Maintained the dose for 28 weeks. Dose gradually decreased over six weeks	Standard treatment control	50 weeks total. Evaluations at baseline, 20 weeks, 39 weeks, and 50 weeks. Final evaluation six weeks after the nicotine was stopped	Motor/primary outcome: no significant difference in primary outcome (UPDRS-III Motor Score Off). Secondary: significant improvements in UPDRS-II (ADLs) and UPDRS-IV (complications) vs. control (p<0.05)
Hauser et al. (2016) [23]	Parkinson's disease. Had symptomatic nOH. Experienced orthostatic dizziness, lightheadedness, or feeling like they were about to faint. 225 patients, ages 18+ years	197 patients received droxidopa; dosage was adjusted over two weeks, then maintained for eight weeks. Dosage ranged from 100 to 600 mg, three times daily	Placebo	10 weeks	Non-motor: droxidopa significantly reduced the fall rate by 77% compared to placebo (p=0.014). Reduced fall-related injuries

Safety Profiles of the Included Interventions

The reviewed interventions demonstrated varied safety profiles, with common adverse events including gastrointestinal issues, dizziness, fatigue, and skin reactions (Table 4). Overall, while most interventions were generally well tolerated, some had significant adverse events requiring careful patient selection and monitoring.

**Table 4:** Summary of safety profile of the included studies. AE: adverse event; Btx: botulinum toxin; CFU: colony forming units; CI: confidence interval; DBS: deep-brain stimulation; HDI: Hamilton Depression Inventory; HOMA-IR: Homeostatic Model Assessment of Insulin Resistance; hs-CRP: high-sensitivity C-reactive protein; iTBS: intermittent theta burst stimulation; ICIQ-SF: International Consultation on Incontinence Questionnaire-Short Form; MDS-UPDRS: Movement Disorder Society-Unified Parkinson's Disease Rating Scale; MMSE: Mini-Mental State Examination; NMSS: Non-Motor Symptoms Scale; OAB-V8: Overactive Bladder-Validated 8-question screening tool; PD: Parkinson's disease; PGIC: Patient Global Impression of Change; QUICKI: quantitative insulin sensitivity check index; RCT: randomized controlled trial; SAE: severe adverse event; SCOPA-COG: Scales for Outcomes in Parkinson's disease-Cognition; SD: standard deviation; SEAPI: Self-Evaluation of Assessing Prostate Cancer International Prostate Symptom Score; TMS: transcranial magnetic stimulation; WMS-III-A: Wechsler Memory Scale-Third Edition-Adult

Studies	Intervention/comparison	Safety profile
Yonguc et al. (2019) [2]	Fesoterodine/placebo	Xerostomia (3.1%), constipation (3.1%); generally well tolerated; AEs were typical of low-dose anticholinergics and limited to mild dry mouth and constipation
Tamtaji et al. (2019) [3]	Probiotics/placebo	Mild gastrointestinal discomfort (bloating, gas) in 16.7%. Very well tolerated; the rate of GI AEs was similar between the probiotic and placebo groups
Athauda et al. (2017) [10]	Exenatide/placebo	High rates of injection site reactions, nausea (51.6%), vomiting, and weight loss. High gastrointestinal burden is expected, but the overall study withdrawal rate was low
Katzenschlager et al. (2018) [11]	Apomorphine/placebo	Common AEs: infusion site nodules (44%), nausea (22%), somnolence (22%). Higher SAE rate (9%), including severe hypotension, myocardial infarction, and cellulitis. High dose modification (48%) and withdrawal (11%) rates
Tröster et al. (2016) [12]	Bilateral subthalamic deep-brain stimulation/delayed activation	Common AEs: cognitive decline (fluency, Stroop), speech disturbance. Key functional concerns include specific post-operative cognitive deficits and psychiatric/mood changes. Primary risks are surgical (e.g., intracranial hemorrhage).
Peball et al. (2020) [13]	Nabilone/placebo	Common AEs: dizziness, somnolence, dry mouth, fatigue. No serious AEs reported; open-label AEs were highly common (77%) but were generally mild and transient
Weintraub et al. (2016) [14]	Rasagiline 1 mg a day/placebo	Common AEs: dizziness, headache, orthostatic hypotension, hallucinations. The withdrawal rate was notably low (two patients) despite reports of rare severe psychiatric AEs (e.g., pathological gambling).
Mamikonyan et al. (2015) [15]	Rivastigmine patch/placebo patch	Common AEs: rash, increased "off" time, GI symptoms. AEs (mainly GI and skin rash) led to a high discontinuation rate (25% vs. 7%) in the active group, indicating tolerance issues
Mestre et al. (2020) [16]	Glycopyrrolate/placebo	High incidence of dry mouth (42.9%), constipation (28.6%), and worsening cognition. Constipation was the most common cause for study withdrawal. Requires close monitoring for anticholinergic effects like urinary retention
Murata et al. (2015) [17]	Zonisamide (25/50 mg)/placebo	Common AEs: somnolence, dyskinesia, constipation. AEs showed a mild, dose-dependent increase (up to 60.9% overall AEs at 50 mg), suggesting good overall tolerability
Simuni et al. (2015) [18]	Pioglitazone/placebo	Common AEs: weight gain (dose-dependent), edema. Safety was generally favorable with no related SAEs; cardiovascular events occurred more frequently in the placebo group
Rieu et al. (2018) [19]	Incobotulinum toxin A/placebo	Common AEs: falls, loss of sensation, localized foot pain. Generally mild, localized AEs related to injection site/muscle weakness, with no unexpected serious events
Trung et al. (2019) [20]	Transcranial magnetic stimulation (TMS)/sham	Mild discomfort at the stimulation site (21.4%). Very safe profile; only minor, transient discomfort reported at the site of stimulation
Villafane et al. (2017) [21]	Transdermal nicotine/standard treatment	High overall AE rate (95%). Severe AE: syncope due to orthostatic hypotension (5%). Close monitoring for hypotension is mandatory
Hauser et al. (2016) [23]	Droxidopa/placebo	Fall-related injuries. Droxidopa was associated with a reduction in fall-related injuries compared to placebo.

Key Adverse Events Between Interventions and Placebo

**Figure 2:**
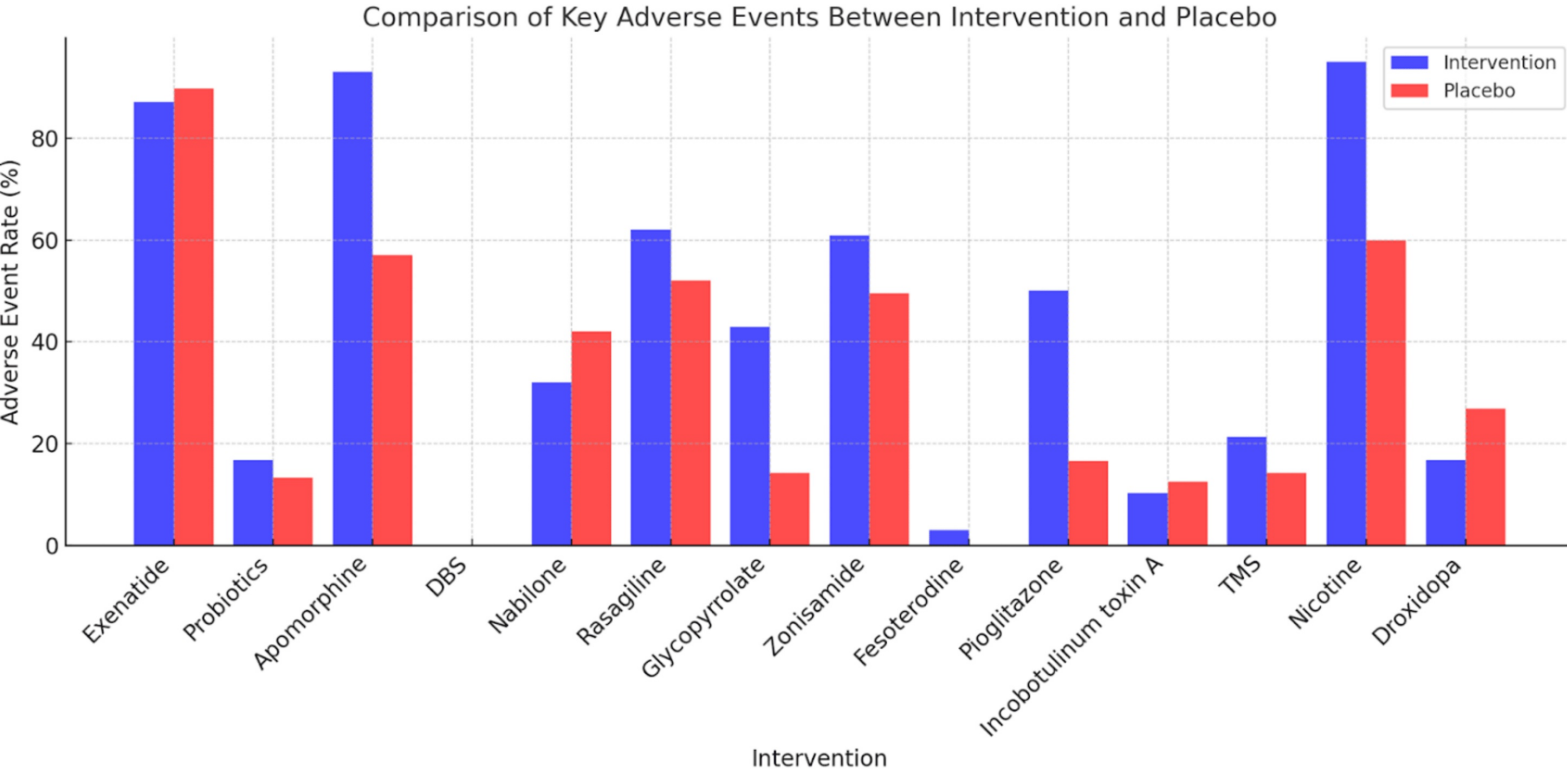
Comparison of key adverse events between intervention and placebo. DBS: deep-brain stimulation; TMS: transcranial magnetic stimulation

Across various interventions compared to placebo, several notable differences in adverse events were observed. Exenatide had higher rates of weight loss, nausea, abdominal pain, and loss of appetite, while the placebo was associated with more sleep disorders, weight gain, and increased dystonia. Probiotics caused mild gastrointestinal discomfort slightly more than the placebo. Apomorphine led to increased skin nodules, nausea, somnolence, and dyskinesia, whereas the placebo had fewer treatment-emergent adverse events. Bilateral subthalamic deep-brain stimulation (DBS) was linked to cognitive decline and mild side effects like dizziness and headaches. Nabilone had more reports of dizziness and dry mouth, while the placebo had more upper respiratory infections. Rasagiline was associated with headaches, orthostatic hypotension, and hallucinations, whereas the placebo had a higher rate of falls. Rivastigmine patch showed increased "off" time and rash, while the placebo had more cognitive decline and depression. Glycopyrrolate led to dry mouth, constipation, and worsened cognition, while the placebo had more urinary frequency. Zonisamide caused more somnolence and dyskinesia compared to placebo. Fesoterodine had reports of dry mouth and constipation, while no adverse events were noted in the placebo group. Pioglitazone was linked to increased adverse events and weight gain, while the placebo saw a decrease in weight. Incobotulinum toxin A increased falls and sensory loss, while the placebo showed more gastroparesis. Transcranial magnetic stimulation (TMS) caused mild stimulation site discomfort, slightly more than sham treatment. Transdermal nicotine was associated with significantly more adverse events, including dizziness and insomnia. Droxidopa resulted in fewer fall-related injuries compared to placebo (Figure 2). Overall, while many interventions presented specific adverse effects, some showed potential benefits over placebo in managing symptoms.

Discussion

This systematic review underscores the potential of various therapeutic strategies targeting cognitive decline and associated symptoms in the management of Parkinson's disease (PD). While the interventions examined showed varying degrees of efficacy and significant heterogeneity in cognitive outcomes, key findings demonstrate promising results - agents such as probiotics and exenatide showed encouraging improvements in motor symptoms and reductions in neuroinflammatory markers. This outcome suggests that managing the non-motor and cognitive aspects of PD could play a crucial role in potentially slowing down the disease's progression and significantly improving patients' overall quality of life.

Efficacy of Therapeutic Strategies on Motor Symptoms and Neuroinflammatory Markers

Despite the primary focus of this review on cognitive decline, several interventions demonstrated noteworthy impacts on motor symptoms and neuroinflammatory markers, highlighting the interconnected nature of PD pathology. For instance, Tamtaji et al. reported a significant decrease in MDS-UPDRS scores within the probiotic group [3]. Specifically, the total score reduction was substantial (difference in mean change: 8.6 points reduction in probiotic vs. placebo group; p=0.01), suggesting that gut microbiota modulation could exert beneficial effects on motor impairments. This was potentially achieved by mitigating systemic inflammation as evidenced by the observed reduction in hs-CRP levels (difference in mean change: 1.7 mg/L reduction in probiotic vs. placebo group; p<0.001). Similarly, Athauda et al. provided compelling evidence that exenatide led to a clinically meaningful improvement in MDS-UPDRS part 3 scores, with a persistent mean difference favoring the intervention over placebo (adjusted difference: -3.5 points; 95% CI: -6.7 to -0.3; p=0.0318) [10]. This motor improvement was further supported by neurobiological markers, with CSF analysis and DaTscan imaging indicating potential neuroprotective effects and a slowing of dopaminergic neuron degeneration. Other interventions also showed positive effects on motor symptoms; for example, Katzenschlager et al. found that apomorphine significantly reduced "off" time and increased "on" time without troublesome dyskinesia [11]. Murata et al. similarly reported a significant reduction in "off" time with zonisamide (50 mg group) [17]. These findings collectively reinforce that interventions, even when targeting broader mechanisms, can yield tangible motor benefits.

Cognitive Outcomes

The impact of the evaluated interventions on cognitive decline in PD presented a more complex and less consistent picture. While some studies showed modest positive trends, others reported minimal or no significant differences compared to placebo, suggesting that cognitive improvement may be highly variable or domain-specific. Weintraub et al. observed that rasagiline was associated with a modest but statistically significant increase in SCOPA-COG scores (difference in mean change: +0.8 points favoring rasagiline), indicating potential cognitive benefits [14]. However, this finding was not uniformly replicated across all cognitive domains, and the MoCA score showed no significant difference between groups. In contrast, Mamikonyan et al., evaluating rivastigmine, found no statistically significant differences in comprehensive cognitive assessments like MoCA or DRS-2 (no statistically significant difference reported between groups), although improvements were noted in the Everyday Cognition Battery, suggesting a nuanced effect on real-world cognitive function [15].

Other interventions, like fesoterodine in a study by Yonguc et al., showed stable cognitive function (MMSE) with no significant difference from placebo [2]. Deep-brain stimulation (DBS) in Tröster et al. was notably associated with declines in specific verbal fluency and Stroop task performance, underscoring potential cognitive trade-offs [12]. Similarly, while Trung et al. found that intermittent theta burst stimulation (iTBS) showed significant improvements in overall cognition and attention, the effects varied across different time points and specific visuospatial abilities [20]. This overall discrepancy in cognitive outcomes is likely attributable to several factors, including the heterogeneity of cognitive assessments used, varying stages of cognitive impairment in the study populations, and the multifaceted nature of cognitive decline in PD.

Safety and Tolerability

The safety and tolerability profiles of the interventions varied considerably across the included studies, as visually represented in Figure 2. While many interventions were generally well-tolerated, some demonstrated a notably higher incidence of adverse events (AEs), necessitating careful patient selection and vigilant monitoring. For instance, interventions like apomorphine, as highlighted by Katzenschlager et al., showed a substantial increase in overall treatment-emergent AEs (93% vs. 57% in placebo) [11]. Similarly, Villafane et al. reported a high rate of AEs (95%) with transdermal nicotine, including severe events like syncope due to orthostatic hypotension [21]. Droxidopa notably resulted in fewer fall-related injuries compared to placebo. It is crucial to acknowledge that while Figure 2 provides an overall comparison of adverse event rates, it does not present statistical measures like relative risks or odds ratios, which limits a precise comparative understanding of safety. Therefore, careful, personalized monitoring for potential side effects remains paramount.

Strengths and Limitations

This systematic review employs several strengths, including the use of RCTs as the primary source of evidence, rigorous quality appraisal using the Cochrane RoB-2 tool [9], and adherence to PRISMA 2020 guidelines [7]. The final included set was restricted to human RCTs, ensuring focus on clinical outcomes.

However, the review faces several limitations primarily stemming from the heterogeneity and reporting quality of the included studies. Methodological constraints included variability in sample sizes and the significant heterogeneity of the therapeutic strategies evaluated, which complicated direct comparison and synthesis. Crucially, data reporting limitations prevented a full quantitative assessment due to the heterogeneity of reported data; standardized effect sizes (e.g., Cohen’s d, odds ratios) could not be calculated, nor could quantitative heterogeneity (I²) or formal subgroup analysis (e.g., early vs. advanced PD) be performed.

Given the reliance on published literature, there is a risk of publication bias, where studies showing significant positive results are more likely to be published than those reporting negative or null findings. Due to the high clinical and methodological heterogeneity of the included interventions (pharmacological, biological, neuromodulatory), formal statistical tests for reporting bias (e.g., funnel plots) were not considered appropriate for this narrative synthesis.

Following RoB-2 assessment, the certainty of evidence for the primary outcomes (motor and cognitive function) was generally assessed as low to moderate. This rating is primarily driven by "some concerns" raised in multiple studies, particularly regarding the risk of bias in the measurement of the outcome (domain 4) and the risk of bias due to missing outcome data (domain 5). The high clinical heterogeneity also contributes to the lower certainty of the overall body of evidence, emphasizing that findings should be interpreted cautiously and should not be overgeneralized.

Additional limitations include the review's scope, which focused primarily on pharmacological and neuromodulatory interventions and did not extensively explore non-pharmacological strategies. Furthermore, the review only included studies published in English, introducing a potential language bias. Finally, the findings may not be fully generalizable to all PD populations. Despite these limitations, this review provides valuable insights into the potential of targeting cognitive decline in PD.

Clinical Implications

The findings from this systematic review offer several important clinical implications for the management of PD. The observed positive effects of interventions like probiotics and exenatide on motor symptoms and neuroinflammatory markers suggest that targeting mechanisms related to gut health and inflammation represents a promising therapeutic avenue for PD. Clinicians may consider exploring these interventions, particularly in patients presenting with prominent motor symptoms or signs of inflammation, as part of a comprehensive management strategy. However, the mixed and often inconsistent results regarding cognitive outcomes emphasize the critical need for a holistic approach that extends beyond any single intervention. Effective PD management must address the diverse motor, non-motor, and cognitive symptoms collectively, recognizing their complex interplay and varied underlying pathologies. Tailoring treatment based on individual patient profiles, symptom constellations, and tolerability is essential.

Future Research

Future research in this critical area should focus on several key directions to advance our understanding and improve patient care. Firstly, larger, multicenter RCTs with standardized protocols are imperative to rigorously validate the efficacy and long-term safety of interventions, particularly those showing promise for cognitive decline in PD. These studies must thoroughly explore the long-term effects of these interventions, not only on cognitive function but also on the overall trajectory of disease progression. Secondly, research should investigate the potential of combination therapies that simultaneously target multiple pathological mechanisms implicated in PD, including neuroinflammation, oxidative stress, protein aggregation, and neurotransmitter imbalances. Thirdly, given the burgeoning evidence for the gut-brain axis's role in PD, future studies should delve deeper into how specific changes in the gut microbiome influence both neuroinflammation and the progression of cognitive decline. Finally, efforts should be directed towards identifying robust biomarkers that can reliably predict treatment response, allowing for a truly personalized medicine approach where interventions are tailored to individual patient characteristics and anticipated benefits, thereby optimizing therapeutic outcomes and minimizing adverse effects.

## Conclusions

This systematic review underscores the potential of various therapeutic strategies targeting cognitive decline and associated symptoms in the management of Parkinson's disease (PD). While the interventions examined showed varying degrees of efficacy and significant heterogeneity in cognitive outcomes, key findings demonstrate promising results as follows: specifically, agents like probiotics and exenatide showed encouraging improvements in motor symptoms and reductions in neuroinflammatory markers. This outcome suggests that managing the non-motor and cognitive aspects of PD could play a crucial role in potentially slowing down the disease's progression and significantly improving patients' overall quality of life.

However, it is vital to acknowledge that PD is a complex, multi-factorial condition, and a single approach is unlikely to be a one-size-fits-all solution. Therefore, future research must prioritize defining the long-term effects of these interventions, rigorously investigating combination therapies that target multiple pathways, and identifying robust biomarkers that can reliably predict individual patient treatment response. Despite the methodological limitations inherent in the heterogeneity of the current literature, this review provides valuable, synthesized insights for both clinicians and researchers. By clarifying the potential benefits and limitations of these targeted interventions, healthcare professionals can make more informed, personalized decisions about treatment strategies, thus paving the way for future innovative approaches to managing PD and improving the lives of those affected by this condition.
